# Supplementary material for: Functional-metabolic coupling in distinct renal cell types coordinates organ-wide physiology and delays premature ageing
Source: Nat Commun. 2023 Dec 18;14:8405. doi: 10.1038/s41467-023-44098-x (PMC10728150; doi:10.1038/s41467-023-44098-x)
Supplement: Supplementary file 1 — Supplementary Information [file 41467_2023_44098_MOESM1_ESM.pdf]

Functional-metabolic coupling in distinct renal cell types coordinates organ-wide physiology and delays premature aging

Supplementary Figure 1

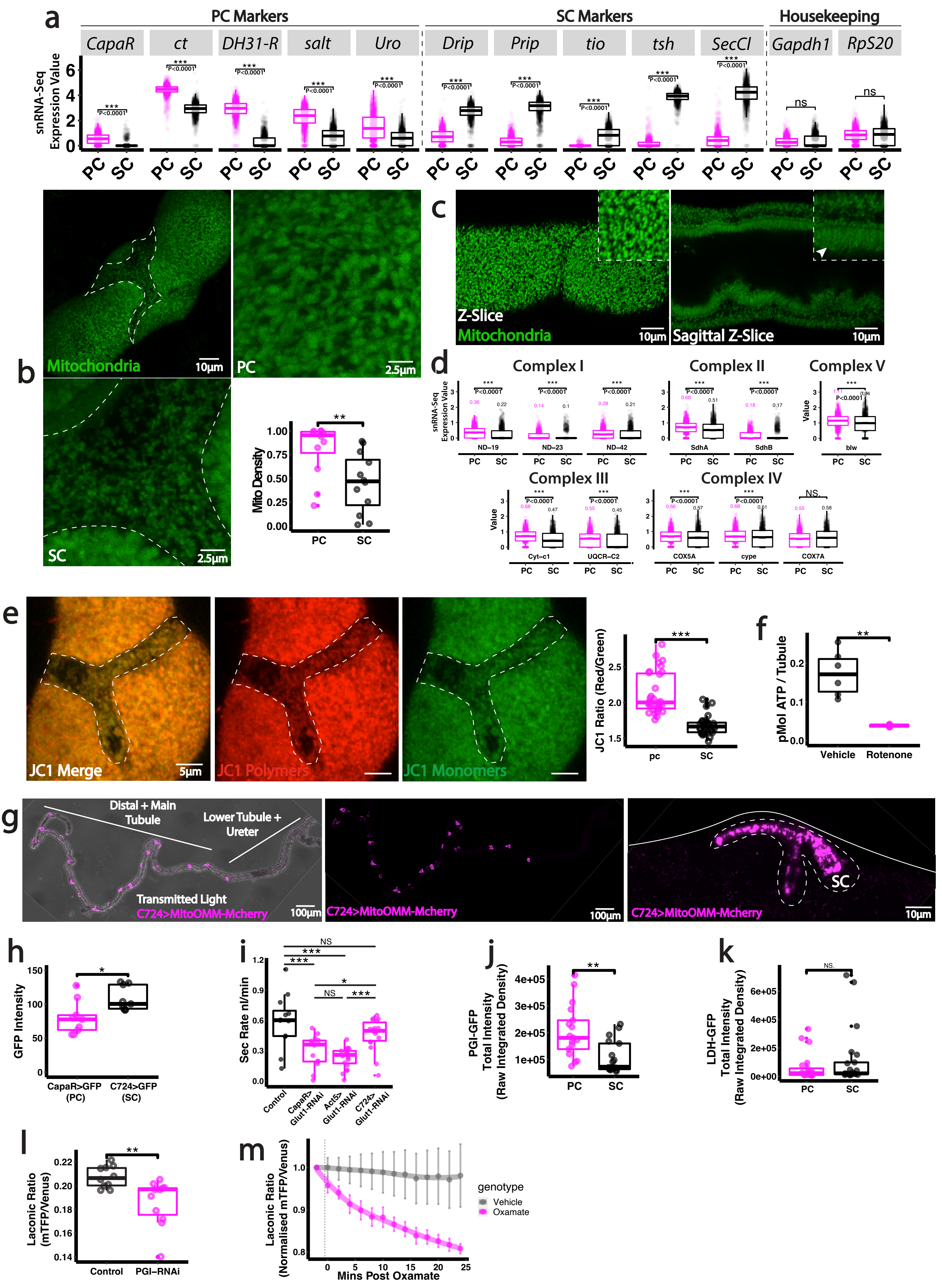

Supplementary Figure 1. PCs and SCs within renal tubules display distinct metabolic profiles.
a Expression of well known PC markers, SC markers and common housekeeping genes in SCs and PCs from publicly available snRNA-seq data (Fly Cell Atlas). **b** Analysis and quantification of mitochondrial content in tubule SCs (dashed line) and PCs (MitoView), zoomed in on PC and SC. **c** Mitochondrial content in PCs, longitudinal and sagittal z-slices (*Act5>MitoGFP*). Arrow indicates mitochondria in apical microvilli. **d** Expression of mitochondrial ETC components in tubule SCs and PCs from publicly available snRNA-Seq data (Fly Cell Atlas). **e** Analysis and quantification of mitochondrial activity (JC1, membrane polarisation) in tubule SCs and PCs. **f** Quantification of ATP in control and rotenone treated tubules, 30 tubules were pooled per replicate. **g** SC specific gal4 driver, C724, driving UAS-mCherry.mito.OMM, with high magnification of SC. **h** *UAS-Cyto-GFP* levels driven by *C724-Gal4* or *CapaR-Gal4*. **i** Secretory activity in control tubules and tubules in which *Act5-Gal4, C724-Gal4 or CapaR-Gal4* drive *Glut1-RNAi*. Total PGI-GFP **j** and LDH-GFP **k** fluorescent intensity in SCs and PCs. **l** Laconic ratio in enterocytes within R2 region of *R2R4>PGI-RNAi* and control guts. **m** Normalised laconic ratio in enterocytes of oxamate and vehicle treated guts over time. Data represented as box and whisker plots (lower and upper hinges correspond to the first and third quartiles, median line within the box, whiskers extend from the hinge to the largest/smallest value, at most 1.5* interquartile range of the hinge) with all data from MpT cells (SCs or PCs) shown as overlaid points. NS = Not Significant, *P<0.05, **P<0.01, ***P<0.001 (unpaired two-tailed t-tests, ANOVA with multiple comparisons or Wilcoxon test with FDR correction where appropriate). *p* values: **b** p=0.00755, **e** p<0.0001, **f** p=0.00232, **h** p=0.01501, **i** Control vs CapaR p=0.000282, Control vs Act5 p<0.0001, CapaR vs C724 p=0.0401, C724 vs Act5 p=0.000453, **j** p=0.00215, **l** p=0.00572. *p* values where p>0.05 labelled as NS, *p* values for **a** and **d** displayed on the Figure. All images representative of >5 tubules. All images are maximum z projections unless otherwise stated. **b** n=12 cells (from 5 tubules) per condition, **e** n=31 cells (from 11 tubules) per condition, **f** n=6 extracts (30 tubules per extract), **h** n=11 tubules per condition, **i** n=12 control, 14 CapaR, 20 Act5 and 18 c724 tubules, **j** 17 cells (from 5 tubules) per condition, **k** n=21 cells (from 3 tubules) per condition, **l** n=10/11 guts per condition, **m** n=4/5 guts per condition. Source data are provided as a Source Data file.

Supplementary Figure 2

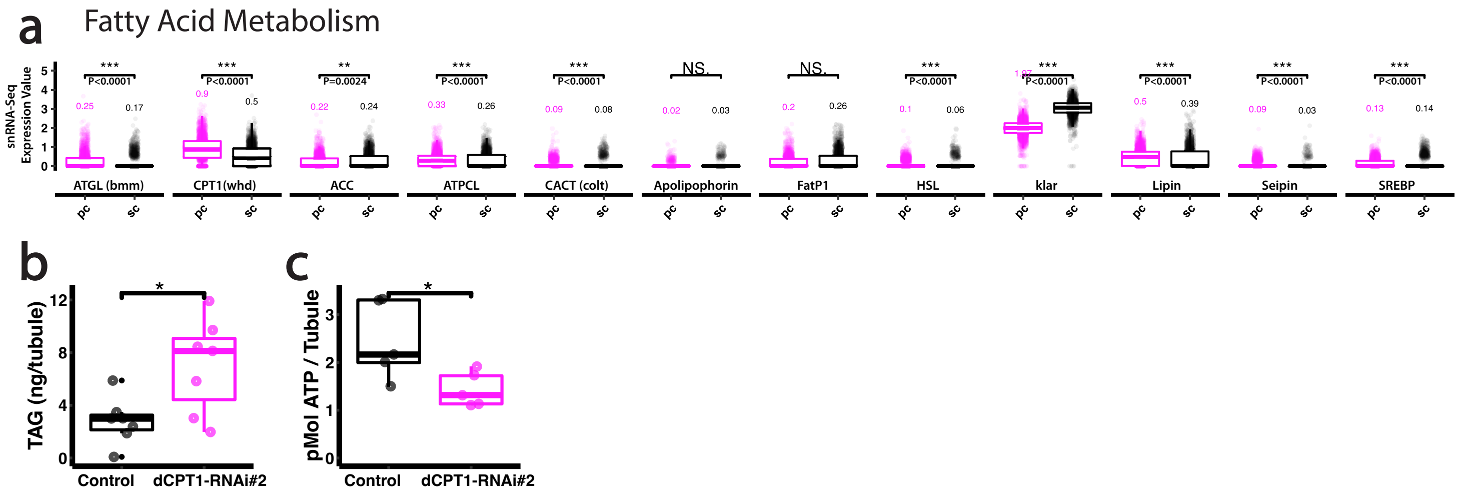

Supplementary Figure 2. Lipid metabolism is enriched in PCs and supports bioenergetic output.
(**a**) Expression of key fatty acid metabolism genes in tubule SCs and PCs from publicly available snRNA-Seq data (Fly Cell Atlas). (**b-c**) Analysis and quantification of triglyceride (TAG, **b**) levels and ATP levels (**c**) in *dCPT1-RNAi* tubules. Data represented as box and whisker plots (lower and upper hinges correspond to the first and third quartiles, median line within the box, whiskers extend from the hinge to the largest/smallest value, at most 1.5* interquartile range of the hinge) with with all data from MpT cells (SCs or PCs) shown as overlaid points. NS = Not Significant, *P<0.05, **P<0.01, ***P<0.001 (unpaired two-tailed t-tests or Wilcoxon test with FDR correction). *p* values: **b** *p*=0.0224, **c** *p*=0.0481. p values where p>0.05 labelled as NS, p values for **a** displayed on the Figure. **b** *n*=7 extracts (20 tubules per extract), **c** *n*=5 extracts (30 tubules per extract). Source data are provided as a Source Data file.

Supplementary Figure 3

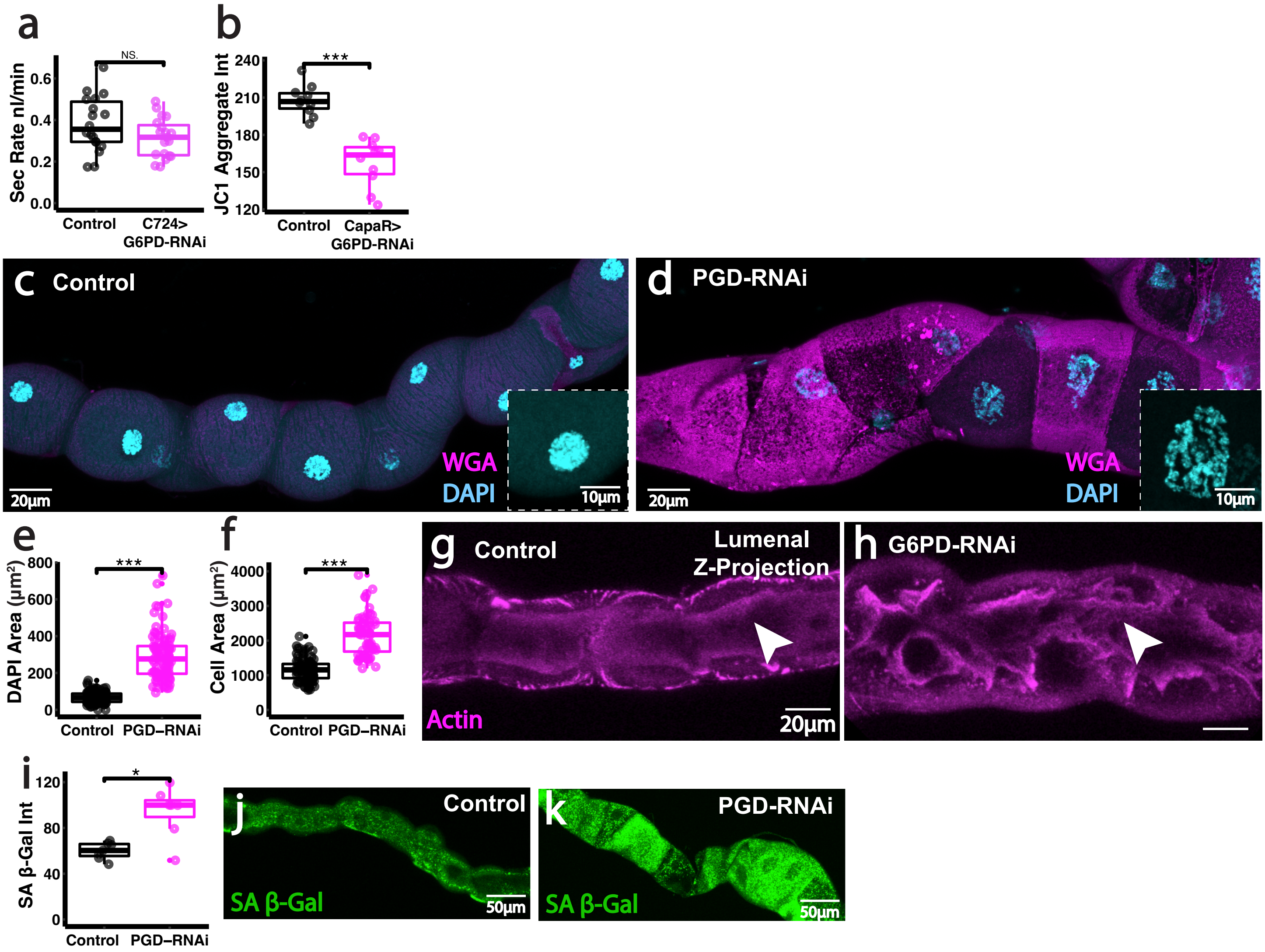

Supplementary Figure 3. Inhibition of PPP activity in PCs causes a senescence-like phenotype.
(**a**) Analysis and quantification of secretion rate in C724>*G6PD-RNAi* tubules. (**b**) Analysis and quantification of mitochondrial activity (JC-1 aggregates, membrane polarisation) in CapaR>*G6PD-RNAi* tubules. (**c-d**) WGA and DAPI staining in *PGD-RNAi* tubules and quantification of nucleus (**e**) and cell (**f**) area. (**g-h**) Z-projection of lumenal Actin (Phalloidin) staining in control and CapaR>*G6PD-RNAi* tubules. (**i-k**) Analysis and quantification of SA β-Gal activity in CapaR>*PGD-RNAi* tubules. Data represented as box and whisker plots (lower and upper hinges correspond to the first and third quartiles, median line within the box, whiskers extend from the hinge to the largest/smallest value, at most 1.5* interquartile range of the hinge) with all data from from MpT main segment sections (b, i), main segment nuclei (e,f) or secretion of individual kidneys (a) shown as overlaid points. NS = Not Significant, *P<0.05, **P<0.01, ***P<0.001 (unpaired two-tailed t-tests). *p* values: **b** *p*<0.0001, **e** *p*<0.0001, **f** *p*<0.0001, **i** *p*=0.0475. *p* values where p>0.05 labelled as NS. For analysis of fluorescent reporters/dyes, two images of different sections of the MpT main segment per fly were imaged. All images representative of >5 tubules. All images are maximum z projections. **a** *n*=18/19 tubules per condition, **b** *n*=10 tubules per condition, **e** *n*=117 nuclei (from 5 control tubules) and 98 nuclei (from 5 RNAi tubules), **f** *n*=87 cells (from 5 control tubules) and 61 cells (from 5 RNAi tubules), **i** *n*=12/13 tubules per condition. Source data are provided as a Source Data file.
