# Supplementary material for: Functional-metabolic coupling in distinct renal cell types coordinates organ-wide physiology and delays premature ageing
Source: Nat Commun. 2023 Dec 18;14:8405. doi: 10.1038/s41467-023-44098-x (PMC10728150; doi:10.1038/s41467-023-44098-x)
Supplement: Supplementary file 3 — Description of Additional Supplementary Files [file 41467_2023_44098_MOESM3_ESM.pdf]

Description of Additional Supplementary Files

Supplementary Movie 1: Time-lapse live imaging of renal PC peroxisomes (labelled using SKL-GFP, green) dynamically interacting with lipid droplets (labelled using LipidTox, magenta) via putative pexapodia. Scale bars represent 0.3µm (left) and 4µm (right). Z-stacks were taken at regular 4 minute (left) or 5 minute (right) time intervals.

Supplementary Movie 2: 3D rendering of confocal image of a renal PC peroxisome (labelled using SKL-GFP, green) interacting with a lipid droplet (labelled using LipidTox, magenta) via putative pexapodia. Scale bar represents 1µm.
