# Supplementary material for: Functional-metabolic coupling in distinct renal cell types coordinates organ-wide physiology and delays premature ageing
Source: Nat Commun. 2023 Dec 18;14:8405. doi: 10.1038/s41467-023-44098-x (PMC10728150; doi:10.1038/s41467-023-44098-x)
Supplement: Supplementary file 6 — Reporting Summary [file 41467_2023_44098_MOESM6_ESM.pdf]

nature portfolio

Corresponding author(s): Helen Weavers

Last updated by author(s): Nov 24, 2023

Reporting Summary

Nature Portfolio wishes to improve the reproducibility of the work that we publish. This form provides structure for consistency and transparency in reporting. For further information on Nature Portfolio policies, see our Editorial Policies and the Editorial Policy Checklist.

Statistics

For all statistical analyses, confirm that the following items are present in the figure legend, table legend, main text, or Methods section.

n/a	Confirmed	
☐	☒	The exact sample size (*n*) for each experimental group/condition, given as a discrete number and unit of measurement
☐	☒	A statement on whether measurements were taken from distinct samples or whether the same sample was measured repeatedly
☐	☒	The statistical test(s) used AND whether they are one- or two-sided<br>*Only common tests should be described solely by name; describe more complex techniques in the Methods section.*
☐	☒	A description of all covariates tested
☐	☒	A description of any assumptions or corrections, such as tests of normality and adjustment for multiple comparisons
☐	☒	A full description of the statistical parameters including central tendency (e.g. means) or other basic estimates (e.g. regression coefficient) AND variation (e.g. standard deviation) or associated estimates of uncertainty (e.g. confidence intervals)
☐	☒	For null hypothesis testing, the test statistic (e.g. *F*, *t*, *r*) with confidence intervals, effect sizes, degrees of freedom and *P* value noted<br>*Give P values as exact values whenever suitable.*
☒	☐	For Bayesian analysis, information on the choice of priors and Markov chain Monte Carlo settings
☒	☐	For hierarchical and complex designs, identification of the appropriate level for tests and full reporting of outcomes
☒	☐	Estimates of effect sizes (e.g. Cohen's *d*, Pearson's *r*), indicating how they were calculated

Our web collection on statistics for biologists contains articles on many of the points above.

Software and code

Policy information about availability of computer code

Data collection	Confocal microscopy data was collected using Leica LAS X software (https://www.leica-microsystems.com/products/microscope-software/p/leica-las-x-ls/downloads/)
Data analysis	Data analysis was performed using ImageJ (2021). All statistical analysis and data visualisation were performed in an R computing environment, with Tidyverse packages (https://www.tidyverse.org/), using rStudio (2021). Mass spec data were searched against the UniProt "Drosophila melanogaster" databases as well as a common contaminants database.

For manuscripts utilizing custom algorithms or software that are central to the research but not yet described in published literature, software must be made available to editors and reviewers. We strongly encourage code deposition in a community repository (e.g. GitHub). See the Nature Portfolio guidelines for submitting code & software for further information.

Data

Policy information about availability of data

All manuscripts must include a data availability statement. This statement should provide the following information, where applicable:

- Accession codes, unique identifiers, or web links for publicly available datasets
- A description of any restrictions on data availability
- For clinical datasets or third party data, please ensure that the statement adheres to our policy

All data underlying the graphs in this study are available in the Source Data Files. For graphical display items throughout the study, raw data points have also been plotted overlaid on each box-and-whisker plot to maximise the reproducibility of the research data.

Fly Cell Atlas single nuclear transcriptomic data is publicly available at https://www.flyrnai.org/scRNA/kidney/ and https://flycellatlas.org/scope. The UniProt "Drosophila melanogaster" database is publicly available at https://www.uniprot.org/proteomes/UP000000803

Research involving human participants, their data, or biological material

Policy information about studies with human participants or human data. See also policy information about sex, gender (identity/presentation), and sexual orientation and race, ethnicity and racism.

Reporting on sex and gender	not applicable
Reporting on race, ethnicity, or other socially relevant groupings	not applicable
Population characteristics	not applicable
Recruitment	not applicable
Ethics oversight	not applicable

Note that full information on the approval of the study protocol must also be provided in the manuscript.

Field-specific reporting

Please select the one below that is the best fit for your research. If you are not sure, read the appropriate sections before making your selection.

☒ Life sciences ☐ Behavioural & social sciences ☐ Ecological, evolutionary & environmental sciences

For a reference copy of the document with all sections, see nature.com/documents/nr-reporting-summary-flat.pdf

Life sciences study design

All studies must disclose on these points even when the disclosure is negative.

Sample size	This study used the fruit fly Drosophila melanogaster. Although Drosophila is an invertebrate species, not covered by the Animal Scientific Procedures Act, we used the minimum number of animals possible to provide rigorous data (ARRIVE guidelines were followed for the study). No a priori sample-size calculation was performed, sample size was set according to the reproducibility of each experiment. The maximum number of replicates were used for each experiment above which additional replicates did not alter the statistical significance.
Data exclusions	No data were excluded from the analyses
Replication	Each experiment was performed with control and test samples processed simultaneously (under equivalent conditions) and repeated in tandem to mitigate spurious non-replicable results. On average each unique experiment was repeated using at least 10 independent biological replicates (where each replicate is tissue obtained from different adult flies) for each experimental group.<br><br>All attempts at replication were successful.
Randomization	Adult flies of the appropriate genotype were randomly selected for analysis
Blinding	In order to avoid bias, all experiments were conducted single-blind where possible. Experimental data generated by microscopy was blinded prior to analysis/quantification (each experimental group were given numbers prior to analysis) and then unblinded after data collection to allow final evaluation.

Reporting for specific materials, systems and methods

We require information from authors about some types of materials, experimental systems and methods used in many studies. Here, indicate whether each material, system or method listed is relevant to your study. If you are not sure if a list item applies to your research, read the appropriate section before selecting a response.

Materials & experimental systems

n/a	Involved in the study
☐	☒ Antibodies
☒	☐ Eukaryotic cell lines
☒	☐ Palaeontology and archaeology
☐	☒ Animals and other organisms
☒	☐ Clinical data
☒	☐ Dual use research of concern
☒	☐ Plants

Methods

n/a	Involved in the study
☒	☐ ChIP-seq
☒	☐ Flow cytometry
☒	☐ MRI-based neuroimaging

Antibodies

Antibodies used	anti-H3K9me3 anti-rabbit Abcam RRID: ab8898 (See https://www.abcam.com/products/primary-antibodies/histone-h3-tri-methyl-k9-antibody-chip-grade-ab8898.html).<br><br>Alexa-488, goat anti-rabbit secondary antibody, RRID: AB_2338046 , Jackson Immuno Research (See https://www.jacksonimmuno.com/catalog/products/111-545-003).
Validation	ab8898: Abcam validation: suitable for: WB, IHC-P, ChIP, ICC/IF. See https://www.abcam.com/products/primary-antibodies/histone-h3-tri-methyl-k9-antibody-chip-grade-ab8898.html<br><br>AAB_2338046: JacksonImmuno validation: Based on immunoelectrophoresis and/or ELISA, the antibody reacts with whole molecule rabbit IgG. See https://www.jacksonimmuno.com/catalog/products/111-545-003

Animals and other research organisms

Policy information about studies involving animals; ARRIVE guidelines recommended for reporting animal research, and Sex and Gender in Research

Laboratory animals	We did not utilise any animals that are covered by the Animal Scientific Procedures Act (1986).<br><br>The following Drosophila melanogaster were used: Act5c-Gal4 (B#4414), ACC-RNAi (VDRC#108631), CapaR-Gal4 (gift from Julian Dow), C724-Gal4 (gift from Barry Denholm), UAS-whd-RNAi #1 (dCPT1, V#105400), UAS-whd-RNAi #2, (dCPT1, B#34066), UAS-ERR-RNAi (B#50686), ERR-GFP (B#38638), UAS-Glut1-RNAi (B#28645), UAS-zw-RNAi (dG6PD, B#50667), GstD1-ARE:GFP (reporter of Nrf2 activity, gift from Ioannis Trougakos), OregonR, UAS-PGI-RNAi (B#51804), UAS-Pex16-RNAi (B#57495), UAS-Pex3RNAi (B#50694), UAS-PGD-RNAi (B#65078), UAS-SKL-GFP (B#28881, B#28881), UAS-mCherry.mito.OMM (B#66532), 4E-BP-dsRed (reporter of ATF4 activity, generated by Kang et al., 201793, gift from Hyung Don Ryoo). Gifts from Irene Miguel-Aliaga: LDH-GFP (generated by Quinones et al., 2007), PGI-GFP (generated by Hudry et al., 2019), Treh-GFP (B#59825), MalA1-GFP (VDRC#318296), R2R4-Gal4, UAS-Laconic11. Drosophila mutants and transgenic lines were obtained from the Bloomington Stock Centre unless otherwise stated. All animals were 7 day old adults unless otherwise stated.
Wild animals	No wild animals were used in the study.
Reporting on sex	not applicable
Field-collected samples	No field collected samples were used in the study
Ethics oversight	The study did not require ethical approval

Note that full information on the approval of the study protocol must also be provided in the manuscript.
